# Dynamical Invariant and Exact Mechanical Analyses for the Caldirola–Kanai Model of Dissipative Three Coupled Oscillators

**DOI:** 10.3390/e23070837

**Published:** 2021-06-30

**Authors:** Salim Medjber, Salah Menouar, Jeong Ryeol Choi

**Affiliations:** 1 Laborarory of Chemical Materials, Department of Physics, Faculty of Sciences, University of Mohamed Boudiaf M’sila, M’sila 28000, Algeria; salim.madjber@univ-msila.dz; 2 Laboratory of Optoelectronics and Compounds (LOC), Departement of Physics, Faculty of Science, University of Ferhat Abbas Setif 1, Setif 19000, Algeria; smenouar@univ-setif.dz; 3 Department of Nanoengineering, Kyonggi University, Yeongtong-gu, Suwon 16227, Korea

**Keywords:** coupled oscillators, unitary transformation, matrix of diagonalization, invariant theory

## Abstract

We study the dynamical invariant for dissipative three coupled oscillators mainly from the quantum mechanical point of view. It is known that there are many advantages of the invariant quantity in elucidating mechanical properties of the system. We use such a property of the invariant operator in quantizing the system in this work. To this end, we first transform the invariant operator to a simple one by using a unitary operator in order that we can easily manage it. The invariant operator is further simplified through its diagonalization via three-dimensional rotations parameterized by three Euler angles. The coupling terms in the quantum invariant are eventually eliminated thanks to such a diagonalization. As a consequence, transformed quantum invariant is represented in terms of three independent simple harmonic oscillators which have unit masses. Starting from the wave functions in the transformed system, we have derived the full wave functions in the original system with the help of the unitary operators.

## 1. Introduction

Description and interpretation of coupled systems are of particular interest in physics because the interaction caused by coupling is responsible for novel quantum effects such as entanglement [1,2] and quadrature squeezing [3]. Coupled oscillatory quantum motions are found in almost all areas of physical sciences, ranging from nanotechnology to biology [4,5,6,7,8]. Coupled oscillatory systems can be used as a model to describe the interactions between atoms in a one-dimensional crystal with spring-like forces under white noise excitations [9,10]. In this regard, oscillatory motions of three masses coupled by four springs were studied analytically by Ndikilar et al. [9]. The dynamics of mixedness and entanglement for three coupled oscillators with arbitrary time-dependent frequencies and coupling parameters has also been investigated [11]. Entanglement and its control in coupled quantum oscillators are crucial for the process of information in quantum cryptography, quantum teleportation, and quantum computing [12].

**Figure 1:**
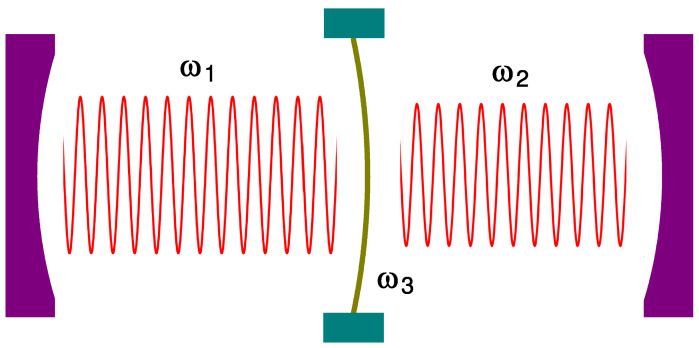
Schematic of a kind of nano-optomechanical three coupled oscillators on which our work can be applied potentially. $\omega_{1}$ and $\omega_{2}$ are frequencies associated with optical modes in cavities, whereas $\omega_{3}$ is a frequency of a mechanical oscillation for a flexible nano membrane. This figure shows the interaction of cavity fields with the nano-mechanical oscillator via the force of radiation pressure.

In particular, coupled oscillator model for nano-optomechanical systems is important because many novel state-of-the-art techniques can be realized by utilizing it. For instance, couplings of photonic systems with mechanical resonators provide fundamental platform for quantum technologies, such as slow/fast-light generation [13], cooling of nanomechanical resonators [14,15], frequency conversion [16,17], and phononic-structure preparation [18]. You can see an example of nano-optomechanical three coupled oscillators from Figure 1.

In this contribution, we will study quantum dynamical invariant for dissipative three coupled oscillators based on exact quantum description of the system. Such invariant can be used in analyzing various mechanical properties of the coupled oscillatory systems [19,20]. For example, quantum mechanics of optomechanical systems can be unfolded by means of the theory of dynamical invariants [20].

There are several methods to calculate dynamical invariants based on algebraic approaches [21,22,23,24,25,26,27]. In the classical case, the Lutzky’s approach [23,24], which relies on the Noether’s theorem is applicable. Some years ago, Bertin et al. developed a new way to calculate dynamical invariants [25], which utilizes the combinations of the classical equations of motion. We also note that there are Lewis-Riesenfeld method for time-dependent Hamiltonian systems [26,27]. In particular, the last method can be flexibly used in both classical and quantum systems. Many research papers were devoted to the construction and applications of dynamical invariants in nonconservative systems [28,29,30]. The method of invariants was also used in the study of three coupled oscillators [31,32,33].

Our paper is structured as follows: In Section 2, we will represent the method for treating three coupled oscillators from preliminary level of mechanics. The results of this work and the related discussion will be placed in Section 3. The classical and quantum invariant quantities for the three coupled oscillators will be derived based on the fundamental Hamiltonian dynamics. The quantum invariant operator will be simplified by a unitary transformation together with a diagonalization through a rotational unitary transformation parameterized by Euler angles. Then, we evaluate the eigenvalues and eigenfunctions of the original invariant operator by taking advantage of the simple expression of the diagonalized invariant operator. We will also derive solutions of the Schrödinger equation of the oscillatory systems using their close relationship with the eigenstates of the invariant operator. Finally, we will conclude our research in Section 4 with some remarks.

## 2. Methods

We start our study by briefly representing how to describe the dynamics of a simple dissipative mechanical oscillatory system. For the linearly damped motion of a 1D oscillatory system subjected to a force field V(X), the Newton’s equation can be written as

(1) $$m\ddot{X}+\gamma \dot{X}=-\vec{\nabla }V\left(X\right).$$

The Lagrangian that produces this motion is of the form

(2) $$L=\exp\left(\delta t\right)\left(\frac{1}{2}m{\dot{X}}^{2}-V\left(X\right)\right),\;\;\;\;\delta =\frac{\gamma }{m},$$

where δ is the damping coefficient. From the well-known relation between the Lagrangian and the Hamiltonian, we can easily have the corresponding Hamiltonian such that

(3) $$H=\frac{P^{2}}{2m}\exp\left(-\delta t\right)+V\left(X\right)\exp\left(\delta t\right).$$

This is the famous Caldirola–Kanai model [34,35], which celebrates 80 years now. This Hamiltonian is dependent on time due to the damping of the system, even if the mass is independent of time.

The above simple mechanical description can be readily extended to coupled oscillators. We consider a system of dissipative three coupled oscillators which have different effective masses ($m_{1},m_{2},m_{3}$), where they are parameterized by the three coordinates $\left(X_{1},X_{2},X_{3}\right)$ [36]. The Hamiltonian of this system can be represented as

(4) $$\begin{array}{ccc} H(t) & = & \frac{1}{2}\sum_{i=1}^{3}\left[\frac{P_{i}^{2}}{e^{\delta t}m_{i}}+e^{\delta t}k_{i}X_{i}^{2}\right]\\ & & +\frac{1}{2}e^{\delta t}\left[k_{12}{\left(X_{1}-X_{2}\right)}^{2}+k_{13}{\left(X_{1}-X_{3}\right)}^{2}+k_{23}{\left(X_{2}-X_{3}\right)}^{2}\right],\; \end{array}$$

where the parameters $k_{i}\left(i=1,2,3\right)$, $k_{12}$, $k_{13}$, and $k_{23}$ are constants; the convention for the lower subscript *i* (including *j* and *k*) given here will be applied throughout the paper. The Hamiltonian in Equation (4) is actually a generalization of 1D single mechanical system expressed by Equation (3). We assumed that damping coefficients for the three oscillators are the same as each other for simplicity; this means that

(5) $$\gamma_{1}/m_{1}=\gamma_{2}/m_{2}=\gamma_{3}/m_{3}\equiv \delta .$$

Let us denote the Poisson bracket for two arbitrary observables $\mu_{i}$ and $\nu_{j}$ as $\left\{\mu_{i},\nu_{j}\right\}$. Then, for canonical local coordinates, we have

(6) $$\left\{X_{i},P_{j}\right\}=\delta_{ij},\;\;\;\;\;\left\{X_{i},X_{j}\right\}=\left\{P_{i},P_{j}\right\}=0.$$

From the use of the Hamilton’s equations, ${\dot{X}}_{i}=\partial H/\partial P_{i}$ and ${\dot{P}}_{i}=-\partial H/\partial X_{i}$, we confirm that the classical equations of motion are given by

(7) $$\left(\begin{array}{c} {\ddot{X}}_{1}\\ {\ddot{X}}_{2}\\ {\ddot{X}}_{3} \end{array}\right)=\left(\begin{array}{c} -\delta {\dot{X}}_{1}\\ -\delta {\dot{X}}_{2}\\ -\delta {\dot{X}}_{3} \end{array}\right)+\left(\begin{array}{ccc} \frac{-\left(k_{1}+k_{12}+k_{13}\right)}{m_{1}} & k_{12}/m_{1} & k_{13}/m_{1}\\ k_{12}/m_{2} & \frac{-\left(k_{2}+k_{12}+k_{23}\right)}{m_{2}} & k_{23}/m_{2}\\ k_{13}/m_{3} & k_{23}/m_{3} & \frac{-\left(k_{3}+k_{13}+k_{23}\right)}{m_{3}} \end{array}\right)\left(\begin{array}{c} X_{1}\\ X_{2}\\ X_{3} \end{array}\right).$$

These equations reveal that coordinates of the system are intricate due to the coupling between mechanical oscillators. Hence, the investigation of mechanical properties of the system is not an easy task. Nevertheless, it may be possible to overcome such a knotty situation by finding a classical invariant quantity of the system. An invariant quantity is an important tool for analyzing mechanical properties of dynamical systems [37,38,39,40]. Let us assume that the formula of the invariant quantity for our system is of the form

(8) $$\begin{array}{ccc} I(t) & = & \frac{1}{2}\sum_{i=1}^{3}\left[\alpha_{i}\left(t\right)P_{i}^{2}+\gamma_{i}\left(t\right)X_{i}^{2}+\beta_{i}\left(t\right)X_{i\;}P_{i}\right]\\ & & +\frac{1}{2}\left[\eta_{12}\left(t\right)X_{1}X_{2}+\eta_{13}\left(t\right)X_{1}X_{3}+\eta_{23}\left(t\right)X_{2}X_{3}\right], \end{array}$$

where $\alpha_{i}\left(t\right)$, $\beta_{i}\left(t\right)$, $\gamma_{i}\left(t\right)$, and $\eta_{ij}\left(t\right)$ are real differentiable functions of time, of which formulae will be evaluated later on.

In fact, the search for such an invariant (a constant of motion) is somewhat difficult for a complicated system. To obtain the invariant quantity by determining the time functions in Equation (8), we use the Liouville’s theorem which is that the phase-space distribution function is constant along the trajectories of the dynamical systems. Via the analysis of the invariant quantity based on this theorem, we can confirm the time evolution of a given dynamical system. Usually, there exist constants of motion in addition to the energy for an integrable dynamical system. These constants for a time-dependent Hamiltonian system do not commute with the Hamiltonian under the Poisson bracket.

## 3. Results and Discussion

### 3.1. Classical Analysis

If we insert Equation (8) with Equation (4) into the Liouville equation

(9) $$\frac{dI}{dt}=\frac{\partial I}{\partial t}+\sum_{i=1}^{3}\left[\frac{\partial I}{\partial X_{i}}\frac{\partial H}{\partial P_{i}}-\frac{\partial I}{\partial P_{i}}\frac{\partial H}{\partial X_{i}}\right]=0,$$

we obtain the coupled differential equations for $\alpha_{i}\left(t\right)$, $\beta_{i}\left(t\right)$, $\gamma_{i}\left(t\right)$, and $\eta_{ij}\left(t\right)$ as

(10) $${\dot{\alpha }}_{i}\left(t\right)=\frac{-2\beta_{i}\left(t\right)}{m_{i}e^{\delta t}},$$

(11) $$\begin{array}{ccc} {\dot{\beta }}_{1}\left(t\right) & = & \left(k_{1}+k_{12}+k_{13}\right)e^{\delta t}\alpha_{1}\left(t\right)-\frac{\gamma_{1}\left(t\right)}{m_{1}e^{\delta t}}, \end{array}$$

(12) $$\begin{array}{ccc} {\dot{\beta }}_{2}\left(t\right) & = & \left(k_{2}+k_{12}+k_{23}\right)e^{\delta t}\alpha_{2}\left(t\right)-\frac{\gamma_{2}\left(t\right)}{m_{2}e^{\delta t}}, \end{array}$$

(13) $$\begin{array}{ccc} {\dot{\beta }}_{3}\left(t\right) & = & \left(k_{3}+k_{13}+k_{23}\right)e^{\delta t}\alpha_{3}\left(t\right)-\frac{\gamma_{3}\left(t\right)}{m_{3}e^{\delta t}}, \end{array}$$

(14) $$\begin{array}{ccc} {\dot{\gamma }}_{1}\left(t\right) & = & 2\left(k_{1}+k_{12}+k_{13}\right)e^{\delta t}\beta_{1}\left(t\right), \end{array}$$

(15) $$\begin{array}{ccc} {\dot{\gamma }}_{2}\left(t\right) & = & 2\left(k_{2}+k_{12}+k_{23}\right)e^{\delta t}\beta_{2}\left(t\right), \end{array}$$

(16) $$\begin{array}{ccc} {\dot{\gamma }}_{3}\left(t\right) & = & 2\left(k_{3}+k_{13}+k_{23}\right)e^{\delta t}\beta_{3}\left(t\right), \end{array}$$

(17) $$\begin{array}{ccc} {\dot{\eta }}_{12}\left(t\right) & = & -2k_{12}\left(t\right)e^{\delta t}\left[\beta_{1}\left(t\right)+\beta_{2}\left(t\right)\right], \end{array}$$

(18) $$\begin{array}{ccc} {\dot{\eta }}_{13}\left(t\right) & = & -2k_{13}\left(t\right)e^{\delta t}\left[\beta_{1}\left(t\right)+\beta_{3}\left(t\right)\right], \end{array}$$

(19) $$\begin{array}{ccc} {\dot{\eta }}_{23}\left(t\right) & = & -2k_{23}\left(t\right)e^{\delta t}\left[\beta_{2}\left(t\right)+\beta_{3}\left(t\right)\right]. \end{array}$$

By solving the above coupled equations, we have

(20) $$\alpha_{i}\left(t\right)=\frac{1}{m_{i}e^{\delta t}}\;,\;\;\;\beta_{i}\left(t\right)=\frac{\delta }{2},$$

(21) $$\gamma_{1}\left(t\right)=\left(k_{1}+k_{12}+k_{13}\right)e^{\delta t},\;\;\;\gamma_{2}\left(t\right)=\left(k_{2}+k_{12}+k_{23}\right)e^{\delta t},\;\;\gamma_{3}\left(t\right)=\left(k_{3}+k_{13}+k_{23}\right)e^{\delta t}\;,$$

(22) $$\eta_{12}\left(t\right)=-2k_{12}e^{\delta t}\;,\;\;\;\eta_{13}\left(t\right)=-2k_{13}e^{\delta t}\;,\;\;\;\;\eta_{23}\left(t\right)=-2k_{23}e^{\delta t}.$$

From the substitution of these outcomes into Equation (8), we have the classical invariant quantity as

(23) $$I\left(t\right)=H\left(t\right)+\frac{\delta }{2}\sum_{i=1}^{3}X_{i}P_{i}.$$

This can be used in analyzing dynamical properties of the oscillatory systems.

Besides the invariant approach that we are interested here, there are other treatments with Hamiltonian for dissipative harmonic oscillators. Bateman carried out a variational approach for the dissipative oscillators with the Langrangian that gives suitable equations of motion of the system [41]. Lemos made the Hamiltonian of the dissipative system simpler through a canonical transformation based on the Hamilton–Jacobi method [42]. Later on, McDonald also proposed another linear (canonical) transformation of position and momentum variables for the damped oscillator, which holds the Liouville’s theorem [43]. These researches may serve as a contrast to the current treatment and would also highlight potential novelties along this line.

In the next section, we will extend this invariant to quantum mechanics, i.e., we will consider its counterpart quantum description in order to unfold the associated quantum theory based on the dynamical invariant.

### 3.2. Quantum Analysis

Due to the analogy of quantum mechanics with the classical mechanics, we can still use the similar notions of an observable and the associated physical state related to our previous description of the system even in a quantum domain. By replacing the canonical variables in the classical invariant with corresponding quantum operators, one gets the quantum invariant operator, such that

(24) $$\hat{I}\left(t\right)=\hat{H}\left(t\right)+\frac{\delta }{4}\sum_{i=1}^{3}\left({\hat{X}}_{i}{\hat{P}}_{i}+{\hat{P}}_{i}{\hat{X}}_{i}\right),$$

where the momentum coordinate is given by ${\hat{P}}_{i}=-i\hbar \frac{\partial }{\partial X_{i}}$. Through the re-interpretation of the classical variables as counterpart quantum operators, Poisson brackets are replaced by commutators $\left[{\hat{X}}_{i},{\hat{P}}_{j}\right]=i\hbar \delta_{ij}$. Then, the analog of Liouville equation in quantum mechanics is the von Neumann one, which is of the form

(25) $$\frac{d\hat{I}\left(t\right)}{dt}=\frac{\partial \hat{I}\left(t\right)}{\partial t}+\frac{1}{i\hbar }\left[\hat{I}\left(t\right),\hat{H}\left(t\right)\right]=0.$$

This can be used to elucidate the time evolution of a quantum state of the system. This consequence stems from the fact that the canonical quantization of the system is possible on the basis of the theorem related to the invariant operator [26,27]. However, for the practical use of the invariant operator for such a purpose, it may be advantageous to transform it to a simple form.

To simplify the invariant, Equation (24), we use the unitary transformation approach. As a first step, we introduce the following transformation of it:

(26) $$\hat{I}={\hat{U}}^{-1}\left(t\right)\hat{I}\left(t\right)\hat{U}\left(t\right),$$

where the unitary operator $\hat{U}\left(t\right)$ is given by [36]

(27) $$\begin{array}{ccc} \hat{U}\left(t\right) & = & \left\{\prod_{i=1}^{3}\exp\left[\frac{i}{2\hbar }\left({\hat{P}}_{i}{\hat{X}}_{i}+{\hat{X}}_{i}{\hat{P}}_{i}\right)\left(\ln\sqrt{m_{i}}+\frac{\delta }{2}t\right)\right]\right\}\\ & & \times \exp\left(-\frac{i\delta }{4\hbar }\sum_{i=1}^{3}{\hat{X}}_{i}^{2}\right). \end{array}$$

Then, after an algebraic evaluation, it is possible to get the transformed invariant as

(28) $$\begin{array}{ccc} \hat{I} & = & \frac{1}{2}\left({\hat{P}}_{1}^{2}+{\hat{P}}_{2}^{2}+{\hat{P}}_{3}^{2}\right)+\frac{1}{2}\left[\frac{k_{1}+k_{12}+k_{13}}{m_{1}}-{\left(\frac{\delta }{4}\right)}^{2}\right]{\hat{X}}_{1}^{2}\\ & & +\frac{1}{2}\left[\frac{k_{2}+k_{12}+k_{23}}{m_{2}}-{\left(\frac{\delta }{4}\right)}^{2}\right]{\hat{X}}_{2}^{2}+\frac{1}{2}\left[\frac{k_{3}+k_{13}+k_{23}}{m_{3}}-{\left(\frac{\delta }{4}\right)}^{2}\right]{\hat{X}}_{3}^{2}\\ & & +\frac{1}{2}\left(\frac{-2k_{12}}{\sqrt{m_{1}m_{2}}}{\hat{X}}_{1}{\hat{X}}_{2}+\frac{-2k_{13}}{\sqrt{m_{1}m_{3}}}{\hat{X}}_{1}{\hat{X}}_{3}+\frac{-2k_{23}}{\sqrt{m_{2}m_{3}}}{\hat{X}}_{2}{\hat{X}}_{3}\right). \end{array}$$

We can confirm that, through this transformation, the invariant operator has been simplified in a way that its ${\hat{P}}_{i}^{2}$ terms are represented in terms of the unit mass. However, the coupling terms which involve $X_{i}X_{j}$ still remain. Due to this, it is still difficult to investigate the basic quantum features of the system in a straightforward way relying on the invariant.

In the next transformation, we will eliminate the coupling terms by diagonalizing the invariant. To this end, we write the invariant in a matrix form by introducing vectors $X={\left(X_{1},X_{2},X_{3}\right)}^{T}$ and $P={\left(P_{1},P_{2},P_{3}\right)}^{T}$ such that

(29) $$\hat{I}=\frac{1}{2}\sum_{i,j=1}^{3}{\hat{P}}_{i}\delta_{ij}{\hat{P}}_{j}+\frac{1}{2}\sum_{i,j=1}^{3}{\hat{X}}_{i}k_{ij}{\hat{X}}_{j},$$

where $k_{ij}$ are *i*th row and *j*th column elements of the matrix

(30) $$k=\left(\begin{array}{ccc} \varpi_{1}^{2} & \frac{1}{2}K_{12} & \frac{1}{2}K_{13}\\ \frac{1}{2}K_{12} & \varpi_{2}^{2} & \frac{1}{2}K_{23}\\ \frac{1}{2}K_{13} & \frac{1}{2}K_{23} & \varpi_{3}^{2} \end{array}\right)\;,$$

while the involved parameters are of the form

(31) $$\begin{array}{ccc} \varpi_{1} & = & \sqrt{\left(k_{1}+k_{12}+k_{13}\right)/m_{1}-{\left(\delta /4\right)}^{2}}, \end{array}$$

(32) $$\begin{array}{ccc} \varpi_{2} & = & \sqrt{\left(k_{2}+k_{12}+k_{23}\right)/m_{2}-{\left(\delta /4\right)}^{2}}, \end{array}$$

(33) $$\begin{array}{ccc} \varpi_{3} & = & \sqrt{\left(k_{3}+k_{13}+k_{23}\right)/m_{3}-{\left(\delta /4\right)}^{2}}, \end{array}$$

(34) $$K_{12}=\frac{-2k_{12}}{\sqrt{m_{1}m_{2}}},\;\;\;K_{13}=\frac{-2k_{13}}{\sqrt{m_{1}m_{3}}},\;\;\;K_{23}=\frac{-2k_{23}}{\sqrt{m_{2}m_{3}}}.$$

In the next section, we will diagonalize the resultant invariant operator, Equation (29), by eliminating the coupling terms.

### 3.3. Rotation Matrix and Diagonalization of Invariant Operator

The diagonalization of the invariant operator $\hat{I}$ can be done by making use of an algebraic approach based on another unitary transformation that corresponds to a 3D rotation. The unitary operator $\hat{\Lambda }$ which will be used to perform a 3D rotation is parameterized by three Euler angles (ϕ,θ,φ).

We consider a rotation matrix of the form

(35) $$R=R_{X_{1}}\left(\phi \right)R_{X_{2}}\left(\theta \right)R_{X_{3}}\left(\varphi \right),$$

where

(36) $$R_{X_{1}}\left(\phi \right)=\left(\begin{array}{ccc} 1 & 0 & 0\\ 0 & c_{\phi } & -s_{\phi }\\ 0 & s_{\phi } & c_{\phi } \end{array}\right),\;\;\;R_{X_{2}}\left(\theta \right)=\left(\begin{array}{ccc} c_{\theta } & 0 & s_{\theta }\\ 0 & 1 & 0\\ -s_{\theta } & 0 & c_{\theta } \end{array}\right),\;\;\;R_{X_{3}}\left(\varphi \right)=\left(\begin{array}{ccc} c_{\varphi } & -s_{\varphi } & 0\\ s_{\varphi } & c_{\varphi } & 0\\ 0 & 0 & 1 \end{array}\right).$$

In Equation (36), we have abbreviated notations as $\left(c_{\zeta },s_{\zeta }\right)\equiv \left(\cos\zeta ,\sin\zeta \right)$ for convenience, while $\zeta \in \left\{\phi ,\theta ,\varphi \right\}$. Note that the rotation matrix R is a real (3×3) orthogonal matrix where its determinant is unity; this can be expressed by an analogous unitary operator (rotation operator) $\hat{\Lambda }\left(t\right)$ in quantum mechanics, such that

(37) $$R\;⟶\;\hat{\Lambda }=\exp\left(i\phi {\hat{J}}_{3}\right)\exp\left(i\theta {\hat{J}}_{2}\right)\exp\left(i\varphi {\hat{J}}_{3}\right),$$

where the three operators, ${\hat{J}}_{1},$${\hat{J}}_{2}$, and ${\hat{J}}_{3}$, are angular momentum generators which are calculated from the definition of the angular momentum $\vec{J}=\vec{X}\times \vec{P}$. Therefore, from the infinitesimal version of Equation (37), we derive the three basic matrices

(38) $${\hat{J}}_{1}=\left(\begin{array}{ccc} 0 & 0 & 0\\ 0 & 0 & -i\\ 0 & i & 0 \end{array}\right),\;{\hat{J}}_{2}=\left(\begin{array}{ccc} 0 & 0 & i\\ 0 & 0 & 0\\ -i & 0 & 0 \end{array}\right),\;{\hat{J}}_{3}=\left(\begin{array}{ccc} 0 & -i & 0\\ i & 0 & 0\\ 0 & 0 & 0 \end{array}\right).$$

These can also be expressed by a single formula

(39) $${\left({\hat{J}}_{k}\right)}_{ij}=-i\hbar \epsilon_{ijk},$$

where $\epsilon_{ijk}$ is the Levi-Civita symbol, which is an antisymmetric tensor. Note that the three matrices ${\hat{J}}_{i}$ satisfy the commutation relation

(40) $$\left[{\hat{J}}_{i},{\hat{J}}_{j}\right]=i\hbar \epsilon_{ijk}{\hat{J}}_{k}.\;\;\;\;\;$$

The switching relationship given in the above equation shows that the operators, ${\hat{J}}_{1}$, ${\hat{J}}_{2}$, and ${\hat{J}}_{3}$, constitute the generators of a Lie algebra with the structure of constants $i\hbar \epsilon_{ijk}$. The associated Lie group is in fact the group of rotation SO(3), which displays the relation between angular momentum operators and the rotation.

We will now see how to diagonalize the invariant $\hat{I}$ by using the matrix representation of the rotation operator. Let us start by writing the matrix k in terms of the new diagonal matrix

(41) $$k=R\;\mathrm{diag}\left[\Omega_{1}^{2},\Omega_{2}^{2},\Omega_{3}^{2}\right]R^{-1}.$$

Then, it is possible to verify the relation $R^{-1}kR=D,$ where

(42) $$D=\mathrm{diag}\left[\Omega_{1}^{2},\Omega_{2}^{2},\Omega_{3}^{2}\right].$$

The new frequencies $\Omega_{i}^{2}$, which represent the eigenvalues of the matrix k, are expressed as [44]

(43) $$\Omega_{1}^{2}=\frac{1}{3}\left(\varpi_{1}^{2}+\varpi_{2}^{2}+\varpi_{3}^{2}\right)+\frac{\Omega^{2}}{3\sqrt{2}}\cos\alpha ,$$

(44) $$\;\;\;\Omega_{2}^{2}=\frac{1}{3}\left(\varpi_{1}^{2}+\varpi_{2}^{2}+\varpi_{3}^{2}\right)+\frac{\Omega^{2}}{3\sqrt{2}}\cos\left(\alpha +\frac{2\pi }{3}\right),$$

(45) $$\;\Omega_{3}^{2}=\frac{1}{3}\left(\varpi_{1}^{2}+\varpi_{2}^{2}+\varpi_{3}^{2}\right)+\frac{\Omega^{2}}{3\sqrt{2}}\cos\left(\alpha -\frac{2\pi }{3}\right),$$

where

(46) $$\Omega^{2}={\left[{\left(\varpi_{1}^{2}-\varpi_{2}^{2}\right)}^{2}+{\left(\varpi_{1}^{2}-\varpi_{3}^{2}\right)}^{2}+{\left(\varpi_{2}^{2}-\varpi_{3}^{2}\right)}^{2}+3\left(K_{12}^{2}+K_{13}^{2}+K_{23}^{2}\right)\right]}^{1/2},$$

(47) $$\alpha =\arccos\left(\frac{A}{2{\left[B\right]}^{3/2}}\right),$$

while

(48) $$\begin{array}{ccc} A & = & -3\left(\varpi_{1}^{2}+\varpi_{2}^{2}\right)\left(\varpi_{1}^{2}+\varpi_{3}^{2}\right)\left(\varpi_{2}^{2}+\varpi_{3}^{2}\right)-27\left(\frac{1}{4}\varpi_{1}^{2}K_{23}^{2}+\frac{1}{4}\varpi_{2}^{2}\;K_{13}^{2}+\frac{1}{4}\varpi_{3}^{2}\;K_{12}^{2}\right)\\ & & +2\left(\varpi_{1}^{6}+\varpi_{2}^{6}+\varpi_{3}^{6}\right)+18\left(\varpi_{1}^{2}\varpi_{2}^{2}\varpi_{3}^{2}+\frac{3}{8}K_{12}K_{13}K_{23}\right)\\ & & +9\left(\varpi_{1}^{2}+\varpi_{2}^{2}+\varpi_{3}^{2}\right)\left(\frac{1}{4}K_{12}^{2}+\frac{1}{4}K_{13}^{2}+\frac{1}{4}K_{23}^{2}\right), \end{array}$$

(49) $$\begin{array}{ccc} B & = & \frac{1}{2}\left[{\left(\varpi_{1}^{2}-\varpi_{2}^{2}\right)}^{2}+{\left(\varpi_{1}^{2}-\varpi_{3}^{2}\right)}^{2}+{\left(\varpi_{2}^{2}-\varpi_{3}^{2}\right)}^{2}\right]\\ & & +\frac{3}{2}\left(\frac{1}{4}K_{12}^{2}+\frac{1}{4}K_{13}^{2}+\frac{1}{4}K_{23}^{2}\right). \end{array}$$

By taking into account the commutation relation $[{\vec{P}}^{2},{\hat{J}}_{i}]=0,$ we can confirm that the expressions of the old conjugate momenta ${\hat{P}}_{i}^{2}$ appeared in the invariant operator $\hat{I}$ remain unchanged. However, the new expressions of the coordinates are given by

(50) $$\begin{array}{ccc} x_{1} & = & X_{1}c_{\theta }c_{\phi }-X_{2}\left(s_{\theta }s_{\varphi }+c_{\theta }c_{\varphi }s_{\phi }\right)+X_{3}\left(c_{\theta }s_{\phi }s_{\varphi }-s_{\theta }s_{\varphi }\right), \end{array}$$

(51) $$\begin{array}{ccc} x_{2} & = & X_{1}s_{\phi }+X_{2}c_{\phi }c_{\varphi }-X_{3}c_{\phi }s_{\varphi }, \end{array}$$

(52) $$\begin{array}{ccc} x_{3} & = & X_{1}c_{\phi }s_{\theta }+X_{2}\left(c_{\theta }s_{\varphi }-s_{\theta }c_{\varphi }s_{\phi }\right)+X_{3}\left(c_{\theta }c_{\varphi }+s_{\theta }s_{\varphi }s_{\phi }\right). \end{array}$$

$p_{i}$ also take similar expressions. From an algebraic evaluation considering these relations, we can show that $\hat{I}$ takes the form

(53) $$\hat{I}=\frac{1}{2}\sum_{i=1}^{3}\left({\hat{p}}_{i}^{2}+\Omega_{i}^{2}{\hat{x}}_{i}^{2}\right).$$

Hence, the invariant $\hat{I}$ corresponds to a sum of three simple harmonic oscillators with unit masses and constant frequencies. Although this simplified invariant has been obtained under the assumption given in Equation (5), it may also be possible to find a simple form of the invariant operator (as in Equation (53)) for the case where the damping coefficients of the three coupled oscillators are not the same each other. Thanks to this simple formula of the transformed invariant, the theory of invariant that we have developed is substantially useful in analyzing the mechanical properties of the system. In subsequent sections, we will show that quantum mechanical analysis of the system is possible by taking advantage of the simplified invariant operator, Equation (53).

### 3.4. Eigenfunctions of the Invariant Operator

As previously mentioned, the invariant quantity is helpful in understanding the quantum dynamics of the system as well as the classical dynamics. To analyze quantum mechanical characteristics of the system, let us see the eigenvalues and eigenfunctions of the invariant operator. In order to obtain them, we introduce creation and annihilation operators in the diagonalized system such that

(54) $${\hat{b}}_{i}=\sqrt{\Omega_{i}/2}{\hat{x}}_{i}+\frac{i}{\sqrt{2\Omega_{i}}}{\hat{p}}_{i},$$

(55) $${\hat{b}}_{i}^{†}=\sqrt{\Omega_{i}/2}{\hat{x}}_{i}-\frac{i}{\sqrt{2\Omega_{i}}}{\hat{p}}_{i}.$$

We easily confirm that these operators obey the usual properties of ladder operators, including the boson canonical commutation rule $[{\hat{b}}_{i},{\hat{b}}_{i}^{†}]=1$. We can also express Equation (53) in terms of ${\hat{b}}_{i}$ and ${\hat{b}}_{i}^{†}$ as

(56) $$\hat{I}=\sum_{i=1}^{3}\hbar \Omega_{i}\left({\hat{b}}_{i}^{†}{\hat{b}}_{i}+\frac{1}{2}\right).$$

Now, let us turn our attention to the invariant operator $\hat{I}\left(t\right)$ in the original system. The formula of $\hat{I}\left(t\right)$ can be obtained from the inverse unitary transformation, which is

(57) $$\hat{I}\left(t\right)=\hat{U}\left(t\right)\hat{I}{\hat{U}}^{-1}\left(t\right).$$

We confirm that the canonical variables are changed through this transformation in a way that

(58) $$\begin{array}{ccc} {\hat{X}}_{i} & ⟶ & \hat{U}\left(t\right){\hat{X}}_{i}{\hat{U}}^{-1}\left(t\right)={\left(m_{i}e^{\delta t}\right)}^{1/2}\hat{X}, \end{array}$$

(59) $$\begin{array}{ccc} {\hat{P}}_{i} & ⟶ & \hat{U}\left(t\right){\hat{P}}_{i}{\hat{U}}^{-1}\left(t\right)={\left(m_{i}e^{\delta t}\right)}^{-1/2}\left({\hat{P}}_{i}+\frac{\delta }{2}m_{i}e^{\delta t}{\hat{X}}_{i}\right). \end{array}$$

Regarding this, the invariant operator $\hat{I}\left(t\right)$, Equation (24), can be readily evaluated to be

(60) $$\hat{I}\left(t\right)=\sum_{i=1}^{3}\hbar \Omega_{i}\left[{\hat{a}}_{i}^{†}\left(t\right){\hat{a}}_{i}\left(t\right)+\frac{1}{2}\right],$$

where ${\hat{a}}_{i}\left(t\right)$ and ${\hat{a}}_{i}^{†}\left(t\right)$ are time-dependent canonical annihilation and creation operators that are defined as

(61) $$\begin{array}{ccc} {\hat{a}}_{i}\left(t\right) & = & \hat{U}\left(t\right){\hat{b}}_{i}{\hat{U}}^{-1}\left(t\right), \end{array}$$

(62) $$\begin{array}{ccc} {\hat{a}}_{i}^{†}\left(t\right) & = & \hat{U}\left(t\right){\hat{b}}_{i}^{†}{\hat{U}}^{-1}\left(t\right). \end{array}$$

It is possible to obtain the full expressions of ${\hat{a}}_{i}\left(t\right)$ and ${\hat{a}}_{i}^{†}\left(t\right)$ using the relations in Equations (58) and (59): we have provided them in Appendix A for convenience.

According to conventional quantum mechanics, the number operators are given by ${\hat{a}}_{i}^{†}{\hat{a}}_{i}$. The eigenvalue equations for them can be written in the form

(63) $${\hat{a}}_{i}^{†}{\hat{a}}_{i}|n_{i},t\rangle =n_{i}|n_{i},t\rangle ,$$

where the eigenvalues, $n_{i}$, are three integer numbers. Because Equation (60) is represented in terms of ${\hat{a}}_{i}^{†}{\hat{a}}_{i}$, the eigenstates of the invariant operator are the same as those of ${\hat{a}}_{i}^{†}{\hat{a}}_{i}$, which are $|n_{i},t\rangle$. Therefore, its normalized eigenstates can be written as a product of the three eigenfunctions of which formulae are derived from the independent zero-point states:

(64) $$\begin{array}{ccc} |n_{1},n_{2},n_{3},t\rangle & = & |n_{1},t\rangle ⊗|n_{2},t\rangle ⊗|n_{3},t\rangle \\ & = & \frac{1}{\sqrt{n_{1}!n_{2}!n_{3}!}}{\left({\hat{a}}_{1}^{†}\right)}^{n_{1}}{\left({\hat{a}}_{2}^{†}\right)}^{n_{2}}{\left({\hat{a}}_{3}^{†}\right)}^{n_{3}}|0,0,0,t\rangle , \end{array}$$

whereas the corresponding eigenvalues are given by

(65) $$\lambda_{n_{1},n_{2},n_{3}}=\sum_{i=1}^{3}\hbar \Omega_{i}\left(n_{i}+\frac{1}{2}\right).$$

On account of the normalization condition, the states in Equation (64) obey

(66) $$\langle n_{1},n_{2},n_{3},t|n_{1}^{\prime },n_{2}^{\prime },n_{3}^{\prime },t\rangle =\delta_{n_{1},n_{1}^{\prime }}\delta_{n_{2},n_{2}^{\prime }}\delta_{n_{3},n_{3}^{\prime }}.$$

In the configuration space, the normalized eigenstates are easily obtained by solving the eigenvalue equation of $\hat{I}$ and they are given by

(67) $$\begin{array}{ccc} & & \langle X_{1},X_{2},X_{3}|n_{1},n_{2},n_{3},t\rangle =\prod_{i=1}^{3}\left[{\left(\frac{\sqrt{\Omega_{i}m_{i}}e^{\delta t/2}}{{\left(\pi \hbar \right)}^{1/2}n_{i}!2^{n_{i}}}\right)}^{1/2}H_{n_{i}}\left(Y_{i}\right)\right]\\ & & \;\;\;\;\;\;\;\;\;\;\;\;\;\;\times \exp\left\{-\sum_{i=1}^{3}\left[\left(\frac{\Omega_{i}}{2\hbar }+\frac{i\delta }{4\hbar }\right)\sum_{j=1}^{3}{\left(R_{ij}\sqrt{m_{j}}e^{\delta t/2}X_{j}\right)}^{2}\right]\right\}, \end{array}$$

where $H_{n}$ are *n*th order Hermite polynomials, $R_{ij}$ are elements of R that correspond to *i*th row and *j*th column, and the functions $Y_{i}$ are represented as

(68) $$\begin{array}{ccc} Y_{1} & = & {\left(\frac{\Omega_{1}e^{\delta t}}{\hbar }\right)}^{1/2}[\sqrt{m_{1}}X_{1}c_{\theta }c_{\phi }-\sqrt{m_{2}}X_{2}\left(s_{\theta }s_{\varphi }+c_{\theta }c_{\varphi }s_{\phi }\right)\\ & & -\sqrt{m_{3}}X_{3}\left(s_{\theta }c_{\varphi }-c_{\theta }s_{\phi }s_{\varphi }\right)], \end{array}$$

(69) $$Y_{2}={\left(\frac{\Omega_{2}e^{\delta t}}{\hbar }\right)}^{1/2}\left[\sqrt{m_{1}}X_{1}s_{\phi }+\sqrt{m_{2}}X_{2}c_{\phi }c_{\varphi }-\sqrt{m_{3}}X_{3}c_{\phi }s_{\varphi }\right],$$

(70) $$\begin{array}{ccc} Y_{3} & = & {\left(\frac{\Omega_{3}e^{\delta t}}{\hbar }\right)}^{1/2}[\sqrt{m_{1}}X_{1}c_{\phi }s_{\theta }+\sqrt{m_{2}}X_{2}\left(c_{\theta }s_{\varphi }-s_{\theta }c_{\varphi }s_{\phi }\right)\\ & & +\sqrt{m_{3}}X_{3}\left(c_{\theta }c_{\varphi }+s_{\theta }s_{\varphi }s_{\phi }\right)]. \end{array}$$

In the next section, we will derive Schrödinger solutions of the system by taking advantage of the eigenstates of $\hat{I}$, which we have obtained here.

### 3.5. The Schrödinger Equation and Its Solutions

The invariant operator and its eigenstates Equation (67) are useful in analyzing the dynamical properties of the system. We will derive the solutions of the Schrödinger equation of the system using their close relationship with the eigenstates of the invariant operator. If we write the time-dependent Schrödinger equation as

(71) $$i\hbar \frac{\partial }{\partial t}|\psi_{n_{1},n_{2},n_{3}}\left(t\right)\rangle =\hat{H}|\psi_{n_{1},n_{2},n_{3}}\left(t\right)\rangle ,$$

its solutions (wave functions) are written in the form

(72) $$|\psi_{n_{1},n_{2},n_{3}}\left(t\right)\rangle =e^{i\zeta_{n_{1},n_{2},n_{3}}\left(t\right)}|n_{1},n_{2},n_{3},t\rangle ,$$

where $\zeta_{n_{1},n_{2},n_{3}}\left(t\right)$ are time-varying phases. From the substitution of Equation (72) into Equation (71), we see that the phases $\zeta_{n_{1},n_{2},n_{3}}\left(t\right)$ satisfy the equation

(73) $$\frac{\partial }{\partial t}\zeta_{n_{1},n_{2},n_{3}}\left(t\right)=\frac{1}{\hbar }\langle n_{1},n_{2},n_{3},t|\left(\frac{\partial }{\partial t}-\hat{H}\right)|n_{1},n_{2},n_{3},t\rangle .$$

A minor evaluation for this equation leads to

(74) $$\zeta_{n_{1},n_{2},n_{3}}\left(t\right)=\sum_{i=1}^{3}\Omega_{i}\left(n_{i}+\frac{1}{2}\right)t.$$

Finally, the wave functions in the configuration space are expressed as

(75) $$\begin{array}{ccc} \langle X_{1},X_{2},X_{3}|\psi_{n_{1},n_{2},n_{3}}\left(t\right)\rangle & = & \langle X_{1},X_{2},X_{3}|n_{1},n_{2},n_{3},t\rangle \\ & & \times \exp\left[-i\sum_{i=1}^{3}\Omega_{i}\left(n_{i}+\frac{1}{2}\right)t\right], \end{array}$$

where the eigenstates $\langle X_{1},X_{2},X_{3}|n_{1},n_{2},n_{3},t\rangle$ are given in Equation (67). Although the wave functions, Equation (75), in the original system are somewhat complicated, they are complete. These wave functions are necessary in evaluating quantum mechanical expectation values of various observables such as position and momentum quadratures, quantum energy, etc. The propagator, Wigner function, and entanglement of the system can also be investigated on the basis of them.

## 4. Conclusions

The dynamical invariant and its diagonalization for dissipative three coupled oscillators were investigated. We also treated the application of the dynamical invariant on quantization of the system. We have constructed the classical invariant quantity at first. Then, it was extended to a quantum one, i.e., we have obtained a rigorous form of the quantum invariant operator using the Liouville-von Neumann equation.

By a unitary transformation, the quantum invariant was transformed to that of a simple coupled oscillatory system which has unit masses. The resultant invariant operator was diagonalized eventually by further transformation using a rotation matrix parameterized by the Euler angles. During the diagonalization procedure, the coupling terms ${\hat{X}}_{i}{\hat{X}}_{j}$ was eliminated and, as a consequence, the invariant reduced to the form of three uncoupled oscillators. The simplicity of the diagonalized quantum invariant is very advantageous in utilizing it in the analysis of the dynamical properties of the system.

The eigenfunctions of the quantum invariant operator were derived in the Fock state by solving its eigenvalue equation. By taking advantage of such eigenfunctions, the wave functions satisfying the Schrödinger equation have been obtained as shown in Equation (75). Such wave functions can be used to characterizing the quantum properties of various coupled oscillatory systems such as nano-optomecanical systems from a fundamental level.

Diagonalizing the invariant operator may have the similar complexity as the diagonalization of the Hamiltonian of the system. Nevertheless, the reason why we are interested in the invariant operator and its diagonalization is that the quantum wave functions associated with a time-dependent Hamiltonian such as Equation (4) are described in terms of the eigenstates of the invariant operator. In our case, the wave functions given in Equation (75) are represented in terms of the eigenstates of $\hat{I}$ given in Equation (67).

We note that the invariant formalism for dynamical systems of which Hamiltonians depend on time admits to obtaining exact classical and quantum solutions without resorting to variational techniques or other approximation manipulations. As a matter of fact, we did not use any approximation in the derivation of (quantum) solutions from the diagonalization of the invariant in this research. This the merit of the approach of complicated dynamical systems based on an invariant, which distinguishes it from other methods in this field.

## Data Availability

Not applicable.

## Appendix A. Annihilation and Creation Operators in the Original System

From straightforward evaluations of Equations (61) and (62) using the formulae of ${\hat{b}}_{i}\left(t\right)$ and ${\hat{b}}_{i}^{†}\left(t\right)$ given in Equations (54) and (55), we have the expressions of the annihilation and creation operators as

(A1) $$\begin{array}{ccc} {\hat{a}}_{1}\left(t\right) & = & {\left(\frac{\Omega_{1}e^{\delta t}}{2}\right)}^{1/2}[\sqrt{m_{1}}X_{1}c_{\theta }c_{\phi }-\sqrt{m_{2}}X_{2}\left(s_{\theta }s_{\varphi }+c_{\theta }c_{\varphi }s_{\phi }\right)\\ & & +\sqrt{m_{3}}X_{3}\left(c_{\theta }s_{\phi }s_{\varphi }-s_{\theta }s_{\varphi }\right)]\\ & & +i{\left(\frac{e^{-\delta t}}{2\Omega_{1}}\right)}^{1/2}[\frac{1}{\sqrt{m_{1}}}\left({\hat{P}}_{1}+\frac{\delta }{2}m_{1}e^{\delta t}{\hat{X}}_{1}\right)c_{\theta }c_{\phi }\\ & & -\frac{1}{\sqrt{m_{2}}}\left({\hat{P}}_{2}+\frac{\delta }{2}m_{2}e^{\delta t}{\hat{X}}_{2}\right)\left(s_{\theta }s_{\varphi }+c_{\theta }c_{\varphi }s_{\phi }\right)\\ & & +\frac{1}{\sqrt{m_{3}}}\left({\hat{P}}_{3}+\frac{\delta }{2}m_{3}e^{\delta t}{\hat{X}}_{3}\right)\left(c_{\theta }s_{\phi }s_{\varphi }-s_{\theta }s_{\varphi }\right)], \end{array}$$

(A2) $$\begin{array}{ccc} {\hat{a}}_{1}^{†}\left(t\right) & = & {\left(\frac{\Omega_{1}e^{\delta t}}{2}\right)}^{1/2}[\sqrt{m_{1}}X_{1}c_{\theta }c_{\phi }-\sqrt{m_{2}}X_{2}\left(s_{\theta }s_{\varphi }+c_{\theta }c_{\varphi }s_{\phi }\right)\\ & & +\sqrt{m_{3}}X_{3}\left(c_{\theta }s_{\phi }s_{\varphi }-s_{\theta }s_{\varphi }\right)]\\ & & -i{\left(\frac{e^{-\delta t}}{2\Omega_{1}}\right)}^{1/2}[\frac{1}{\sqrt{m_{1}}}\left({\hat{P}}_{1}+\frac{\delta }{2}m_{1}e^{\delta t}{\hat{X}}_{1}\right)c_{\theta }c_{\phi }\\ & & -\frac{1}{\sqrt{m_{2}}}\left({\hat{P}}_{2}+\frac{\delta }{2}m_{2}e^{\delta t}{\hat{X}}_{2}\right)\left(s_{\theta }s_{\varphi }+c_{\theta }c_{\varphi }s_{\phi }\right)\\ & & +\frac{1}{\sqrt{m_{3}}}\left({\hat{P}}_{3}+\frac{\delta }{2}m_{3}e^{\delta t}{\hat{X}}_{3}\right)\left(c_{\theta }s_{\phi }s_{\varphi }-s_{\theta }s_{\varphi }\right)], \end{array}$$

(A3) $$\begin{array}{ccc} {\hat{a}}_{2}\left(t\right) & = & {\left(\frac{\Omega_{2}e^{\delta t}}{2}\right)}^{1/2}\left[\sqrt{m_{1}}X_{1}s_{\phi }+\sqrt{m_{2}}X_{2}c_{\phi }c_{\varphi }-\sqrt{m_{3}}X_{3}c_{\phi }s_{\varphi }\right]\\ & & +i{\left(\frac{e^{-\delta t}}{2\Omega_{2}}\right)}^{1/2}[\frac{1}{\sqrt{m_{1}}}\left({\hat{P}}_{1}+\frac{\delta }{2}m_{1}e^{\delta t}{\hat{X}}_{1}\right)s_{\phi }\\ & & +\frac{1}{\sqrt{m_{2}}}\left({\hat{P}}_{2}+\frac{\delta }{2}m_{2}e^{\delta t}{\hat{X}}_{2}\right)c_{\phi }c_{\varphi }\\ & & -\frac{1}{\sqrt{m_{3}}}\left({\hat{P}}_{3}+\frac{\delta }{2}m_{3}e^{\delta t}{\hat{X}}_{3}\right)c_{\phi }s_{\varphi }], \end{array}$$

(A4) $$\begin{array}{ccc} {\hat{a}}_{2}^{†}\left(t\right) & = & {\left(\frac{\Omega_{2}e^{\delta t}}{2}\right)}^{1/2}\left[\sqrt{m_{1}}X_{1}s_{\phi }+\sqrt{m_{2}}X_{2}c_{\phi }c_{\varphi }-\sqrt{m_{3}}X_{3}c_{\phi }s_{\varphi }\right]\\ & & -i{\left(\frac{e^{-\delta t}}{2\Omega_{2}}\right)}^{1/2}[\frac{1}{\sqrt{m_{1}}}\left({\hat{P}}_{1}+\frac{\delta }{2}m_{1}e^{\delta t}{\hat{X}}_{1}\right)s_{\phi }\\ & & +\frac{1}{\sqrt{m_{2}}}\left({\hat{P}}_{2}+\frac{\delta }{2}m_{2}e^{\delta t}{\hat{X}}_{2}\right)c_{\phi }c_{\varphi }\\ & & -\frac{1}{\sqrt{m_{3}}}\left({\hat{P}}_{3}+\frac{\delta }{2}m_{3}e^{\delta t}{\hat{X}}_{3}\right)c_{\phi }s_{\varphi }], \end{array}$$

(A5) $$\begin{array}{ccc} {\hat{a}}_{3}\left(t\right) & = & {\left(\frac{\Omega_{3}e^{\delta t}}{2}\right)}^{1/2}[\sqrt{m_{1}}X_{1}c_{\phi }s_{\theta }+\sqrt{m_{2}}X_{2}\left(c_{\theta }s_{\varphi }-s_{\theta }c_{\varphi }s_{\phi }\right)\\ & & +\sqrt{m_{3}}X_{3}\left(c_{\theta }c_{\varphi }+s_{\theta }s_{\varphi }s_{\phi }\right)]\\ & & +i{\left(\frac{e^{-\delta t}}{2\Omega_{3}}\right)}^{1/2}[\frac{1}{\sqrt{m_{1}}}\left({\hat{P}}_{1}+\frac{\delta }{2}m_{1}e^{\delta t}{\hat{X}}_{1}\right)c_{\phi }s_{\theta }\\ & & +\frac{1}{\sqrt{m_{2}}}\left({\hat{P}}_{2}+\frac{\delta }{2}m_{2}e^{\delta t}{\hat{X}}_{2}\right)\left(c_{\theta }s_{\varphi }-s_{\theta }c_{\varphi }s_{\phi }\right)\\ & & +\frac{1}{\sqrt{m_{3}}}\left({\hat{P}}_{3}+\frac{\delta }{2}m_{3}e^{\delta t}{\hat{X}}_{3}\right)\left(c_{\theta }c_{\varphi }+s_{\theta }s_{\varphi }s_{\phi }\right)], \end{array}$$

(A6) $$\begin{array}{ccc} {\hat{a}}_{3}^{†}\left(t\right) & = & {\left(\frac{\Omega_{3}e^{\delta t}}{2}\right)}^{1/2}[\sqrt{m_{1}}X_{1}c_{\phi }s_{\theta }+\sqrt{m_{2}}X_{2}\left(c_{\theta }s_{\varphi }-s_{\theta }c_{\varphi }s_{\phi }\right)\\ & & +\sqrt{m_{3}}X_{3}\left(c_{\theta }c_{\varphi }+s_{\theta }s_{\varphi }s_{\phi }\right)]\\ & & -i{\left(\frac{e^{-\delta t}}{2\Omega_{3}}\right)}^{1/2}[\frac{1}{\sqrt{m_{1}}}\left({\hat{P}}_{1}+\frac{\delta }{2}m_{1}e^{\delta t}{\hat{X}}_{1}\right)c_{\phi }s_{\theta }\\ & & +\frac{1}{\sqrt{m_{2}}}\left({\hat{P}}_{2}+\frac{\delta }{2}m_{2}e^{\delta t}{\hat{X}}_{2}\right)\left(c_{\theta }s_{\varphi }-s_{\theta }c_{\varphi }s_{\phi }\right)\\ & & +\frac{1}{\sqrt{m_{3}}}\left({\hat{P}}_{3}+\frac{\delta }{2}m_{3}e^{\delta t}{\hat{X}}_{3}\right)\left(c_{\theta }c_{\varphi }+s_{\theta }s_{\varphi }s_{\phi }\right)]. \end{array}$$
